# Does the Restoration Design and Material Affect Indirect Restorations' Marginal and Internal Gap, Interfacial Volume, and Fatigue Behavior?

**DOI:** 10.1055/s-0045-1802569

**Published:** 2025-03-12

**Authors:** Gabriel Kalil Rocha Pereira, Rafaela Oliveira Pilecco, Lucas Saldanha da Rosa, Renan Vaz Machry, Andrea Baldi, Nicola Scotti, Luiz Felipe Valandro, João Paulo Mendes Tribst, Cornelis Johannes Kleverlaan

**Affiliations:** 1 Department of Restorative Dentistry, Faculty of Dentistry, Universidade Federal de Santa Maria (UFSM), Santa Maria, Rio Grande do Sul State, Brazil; 2 Department of Conservative Dentistry, Faculty of Dentistry, Federal University of Rio Grande do Sul (UFRGS), Porto Alegre, Rio Grande do Sul State, Brazil; 3 Department of Restorative Dentistry, Faculty of Dentistry, Federal University of Minas Gerais (UFMG), Belo Horizonte, Minas Gerais State, Brazil; 4 Department of Surgical Sciences, Dental School, University of Turin, Turin, Italy; 5 Department of Reconstructive Oral Care, Academic Centre for Dentistry Amsterdam (ACTA), Universiteit van Amsterdam and Vrije Universiteit, Amsterdam, North Holland, the Netherlands; 6 Department of Dental Materials Science, Academic Centre for Dentistry Amsterdam (ACTA), Universiteit van Amsterdam and Vrije Universiteit, Amsterdam, North Holland, the Netherlands

**Keywords:** ceramics, composite resins, dental marginal adaptation, mechanical tests, finite element analysis

## Abstract

**Objectives:**

This article evaluates the marginal and internal gap, interfacial volume, and fatigue behavior in computer-aided design-computer-aided manufacturing (CAD-CAM) restorations with different designs (crowns or endocrowns) made from lithium disilicate-based ceramic (LD, IPS e.max CAD, Ivoclar AG) or resin composite (RC, Tetric CAD, Ivoclar AG).

**Materials and Methods:**

Simplified LD and RC crowns (-C) and endocrowns (-E) were produced ( *n* = 10) using CAD-CAM technology, through scanning (CEREC Primescan, Dentsply Sirona) and milling (CEREC MC XL, Dentsply Sirona), and then adhesively bonded to fiberglass-reinforced epoxy resin. Computed microtomography was used to assess the marginal and internal gap and interfacial volume. A cyclic fatigue test (20 Hz, initial load = 100 N/5,000 cycles; step-size = 50 N/10,000 cycles until 1,500 N, if specimens survived, the step-size = 100 N/10,000 cycles until failure) was performed. Topography, finite element analysis (FEA), and fractography were also executed.

**Statistical analysis:**

Two-way analysis of variance and Tukey's post hoc tests were employed ( *α* = 0.05) for marginal and internal gap and interfacial volume. Survival analysis based on Kaplan–Meier and Mantel–Cox tests ( *α* = 0.05) was used for fatigue data.

**Results:**

RC crowns demonstrated the smallest marginal gap, LD crowns the largest. Endocrowns presented intermediary marginal gap values. Internal gaps were all above the planned 120 µm space. The lowest gap was seen at the cervical-axial angle at crowns, regardless of material. At the axio-occlusal angle, LD crowns presented a lower gap than RC; meanwhile, there was no difference among endocrowns. When comparing occlusal/pulpal space, LD crowns showed the lowest values, and RC-C, LD-E, and RC-E were statistically similar. Fatigue testing revealed superior behavior for RC restorations, withstanding higher loads and more cycles before failure compared to LD. FEA indicated that the crowns required higher stress concentration to unleash their failure than endocrowns. Fractographic features confirm failure origin at surface defects located at the restoration/cement intaglio surface, where it concentrated the highest maximum principal stress.

**Conclusion:**

RC crowns and endocrowns presented lower marginal gaps than LD ones. Differences in other internal gap outcomes exist but within a nonclinically relevant threshold. The restoration fatigue behavior was influenced by the CAD-CAM material, but not by its design.

## Introduction

When restoring endodontically treated teeth, clinicians need to select the restoration design and material to achieve long-lasting oral rehabilitations. It is known that one of the main precursors for the longevity of the treatment is the maintenance of remnant tooth tissue, which many situations today challenge the clinician between choosing to rehabilitate with a full-coverage crown, usually associated with an intraradicular post, or an endocrown. 1 2 3

Digital dentistry workflow has become a daily routine in many clinical practices worldwide, employing computer-aided design-computer-aided manufacturing (CAD-CAM) systems to efficiently deliver predictable long-lasting restorations. 4 5 6 In this sense, the use of CAD-CAM lithium disilicate-based ceramics (LD) and resin composite blocks (RC) are widespread options, based on their inherent adequate properties in mechanical, functional, optical, and aesthetical aspects. 7 8 LD ceramics are characterized by crystal particle-filled glass, whereas the crystalline content acts as a reinforcing arrangement within the glass' main structure. 9 The clinical longevity of CAD-CAM LD crowns is already shown to be adequate, above 80% survival rate, on follow-ups over 15 years. 7 Similarly, endocrowns also showed survival rates above 80% on a 10-year follow-up. 3 Indirect RC restorations are also already validated as a long-lasting material. 10 11 However, the performance of CAD-CAM resin-based restorations remains more challenging and is still under discussion, with a higher risk for complications in the short term (up to 3 years). 12 Despite that, their indications are being explored and their use has been intensified based on their inherent properties of more compatible elastic modulus to the tooth structure, lesser potential for antagonist wear, and easier fabrication and repair than dental ceramics. 13 14

Few studies compare differences in restoration design (e.g. crown or endocrown) and restoration material (e.g. CAD-CAM LD or RC) in terms of marginal gap, internal gap, interfacial volume, and fatigue behavior. 15 16 17 Literature supports that marginal gaps should be less than 120 μm for clinically acceptable performance. 18 Despite that, the American Dental Association recommends that the luting thickness should not exceed 40 μm. 19 It becomes clear the lack of consensus in the literature regarding which gap limit is acceptable from a clinical standpoint, but it is consensual that increased gaps, in other words, poor adaptation of restoration, can predispose the patient to poor periodontal health maintenance, 20 facilitate dissolution of the luting agent, and trigger a worse load distribution of the restorative set. 21 Another important aspect directly related to the gap size between restoration and the tooth substrate is the thickness of the luting agent. It is known that thicker intaglio surfaces are detrimental to the performance of the luting agent, increasing the risk of bubble occurrence, which can act as trigger points for stress concentration and restoration fracture. 21 22 23 24 Summed, there is also the fact that CAD-CAM milling has also been known to induce surface/subsurface damage and residual stresses, which could favor restoration fracture. 25 26 27 28

Based on the aforementioned presupposes, it becomes clear the need for more studies that compare and characterize the performance of different restoration designs and materials, on marginal and internal gaps, and interfacial volume of the bonding interface, and correlate those outcomes to the mechanical fatigue behavior of such restorations. Thus, this study aims to evaluate the marginal gap, internal gap, interfacial volume, and fatigue behavior of CAD-CAM restorations with different designs (endocrowns or crowns) made of different CAD-CAM materials (lithium disilicate-based ceramic [LD], or resin composite [RC]). Regarding the scarce existence of guiding literature on the theme this study adopted the null hypothesis that: the marginal gap, internal gap, and interfacial volume would not be affected by the restoration design (1); and by the restoration material (2). Additionally, it was pondered that the fatigue behavior of such restorations also would not be affected by both factors (hypotheses 3 and 4, respectively).

## Material and Methods

**Fig. 1:**
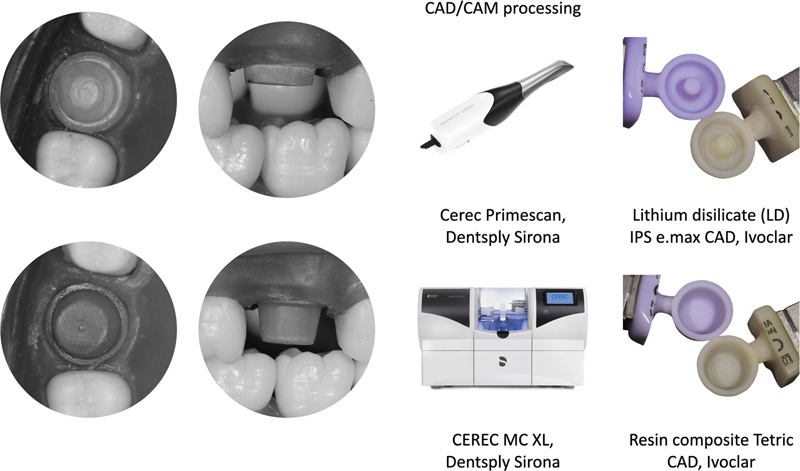
Illustration of the flow for specimen manufacturing, from the fiberglass-reinforced epoxy resin dies, simulating a tooth preparation for crown or endocrown, positioning on the typodont model, its scanning and milling, to obtaining the final restoration.

The study design and materials used are described in Table 1 . An illustration of the study flow, from specimen manufacturing to positioning on a typodont model (AC 103 model, Pronew Odonto, São Gonçalo, Brazil), scanning, milling, and obtaining the final restoration, is shown in Fig. 1 .

**Table 1:** Study design and materials

Group code	Restorative design	Material	Analysis
**LD-C**	Crown	Lithium disilicate (IPS e.max CAD, Ivoclar AG)	- Marginal and internal gaps, interfacial volume using uCT - Fatigue behavior - Topography and fractography via SEM - Finite element analysis
**RC-C**		Resin composite (Tetric CAD, Ivoclar AG)	
**LD-E**	Endocrown	Lithium disilicate	
**RC-E**		Resin composite	

Abbreviations: C, crown; CAD, computer-aided design; E, endocrown; LD, lithium disilicate; uCT, computed microtomography; RC, resin composite; SEM, scanning electron microscopy.

Fiberglass-reinforced epoxy resin rods (10 mm diameter, Protec Produtos Técnicos Ltda., São Paulo, Brazil) were milled into dies using a lathe (Diplomat 3001; Nardini, Americana, Brazil) to simulate simplified tooth preparations for a crown or an endocrown. 15 For crowns, the tooth preparation presented a conical shape with axial walls at an inclination of 8 degrees, and a uniform occlusal and axial space of 1.5 mm, the height of the final tooth preparation was 5.32 mm. 25 For endocrowns, the same external dimensions were used, but the differences were that the height of the final tooth preparation was set as 2 mm, and there was a deepening in the center of the occlusal surface with 4 mm deep, simulating the intrapulpal preparation. The axial walls of such entrance presented the same 8-degree inclination of the external axial wall, and the thickness of the axial wall was set at 2 mm. 15

For restoration digital planning, each fiberglass-reinforced epoxy resin die was settled into a typodont model ( Fig. 1 ), and then, using the CEREC Primescan intraoral scanner (Dentsply Sirona, Charlotte, United States), the preparation was digitalized and the restorations were designed individually in the design software (CEREC 4.5.2, Dentsply Sirona). Simplified crowns ( *n* = 10) were planned with a 1.2-mm thickness at the occlusal surface, which was designed flat, and a cement space of 120 µm, according to the manufacturer's standard instructions. Meanwhile, simplified endocrowns ( *n* = 10) were planned with the same design, but the occlusal thickness was set at 1.5 mm, which is the minimal thickness preconized for this design by the manufacturer of the used restorative materials (Ivoclar AG, Schaan, Liechtenstein). Both crowns and endocrowns were wet milled in a 4-axis machine with brand new set of 2 burs each (Step bur 12S and Cylinder pointed bur 12S) at 42,000 revolutions per minute (rpm) (CEREC MC XL, Dentsply Sirona), considering the two restorative materials (lithium disilicate-based ceramic, LD, IPS e.max CAD, Ivoclar AG; or resin composite, RC, Tetric CAD, Ivoclar AG). Milling was done according to the manufacturer's standards. Subsequently, LD restorations were crystallized in a furnace according to the manufacturer's instructions (speed crystallization in Programat CS4, Ivoclar AG), and RC ones remained untouched. Each restoration was tested into its corresponding die before bonding procedures, to guarantee optimal setting.

After, the restorations and dies were cleaned in an ultrasonic bath with 70% alcohol for 5 minutes and bonded according to the manufacturer's guidelines and considering each substrate's intrinsic composition. The epoxy dies were treated with approximately equal to 5% hydrofluoric acid (IPS Ceramic Etching Gel, Ivoclar AG) for 60 seconds, followed by air-water rinsing for 30 seconds, air-drying, and an active application for 20 seconds of an adhesive (Adhese Universal, Ivoclar AG), which was not light-cured. LD restorations were etched with approximately equal to 5% hydrofluoric acid for 20 seconds, followed by air-water rinsing for 30 seconds, followed by the active application of a silane-based primer (Porcelain Silane, B.J.M. Laboratories Ltd, Or Yehuda, Israel) for 15 seconds, maintained reacting for 45 seconds, and then air-dried. RC restorations were air-abraded with aluminum oxide powder (50 µm at 10 mm distance, 1 bar pressure; Ossido di Alluminio, Henry Schein, New York, United States), and then received an adhesive application (Adhese Universal, Ivoclar AG), as descripted previously. Lastly, each restoration was adhesively bonded in each respective epoxy die using a dual cure resin cement (Variolink Esthetic DC, Ivoclar AG) under a standardized load (500 gr) in a specific device. All resin cement excess was removed, and a light unit (Starlight Uno, Mectron, Carasco, Italy) with over 1,500 mW/cm ^2^ power, and 440 to 465 nm light wavelength, was used to cure the resin cement for 40 seconds in each direction (0, 90, 180, and 270 degrees, and on the top).

Marginal gap, internal gap, and interfacial volume assessments of each sample were made using a computed microtomography analysis (SkyScan 1172 Micro-CT, Bruker, Billerica, United States) with 100 Kv, 100 µA, source–object distance = 89.510 mm, source–detector distance = 217.578 mm, pixel binning = 9.01 µm, exposure time/projection = 846 ms, aluminum and copper (Al + Cu) filter, pixel size = 14.83 µm, averaging = 5, and rotation step = 0.6 degrees. 29 The images were obtained using NRecon software (Bruker) with different parameters for each restorative material, LD/RC, respectively, as follows: smoothening = 0/2, misalignment compensation = 5/4.5, ring artifacts reduction = 10/2, and beam-hardening correction = 30/40%. Finally, the images were uploaded onto the Data Viewer software (Bruker), and three sagittal and coronal slices were randomly selected in each sample to be analyzed at ImageJ 1.53t (National Institutes of Health, Bethesda, United States) to obtain the marginal and internal gap values. 30 For crowns a single vertical measurement was made to define the marginal gap, while three different locations were considered for the internal gap, resulting in a total of 42 measurements per specimen. Meanwhile, for endocrowns, two regions of interest (ROIs) for the marginal gap were considered, and three ROIs for the internal gap, resulting in 54 measurements per specimen. Additionally, through a three-dimensional method, in a specific software (Mimics Medical, v. 23.0; Materialise, Leuven, Belgium), the volumetric measurement of the resin cement layer of each restoration was evaluated by checking the dimensions of the bonding interface. Volumetric calculation of the resultant mask was collected in mm ^3^ and statistically analyzed. 31

For assessing mechanical fatigue behavior using an electric mechanical testing machine (Instron ElectroPuls E3000, Instron, Norwood, United States), each restorative set was positioned onto a base, submerged onto distilled water, and cyclic loading was applied with a stainless steel hemispherical piston (Ø = 40 mm), positioned in the center of the occlusal surface of the specimen. 32 An adhesive tape (110 µm) was interposed between the piston and specimen. Using a frequency of 20 Hz, an initial load of 100 N for 5,000 cycles and incremental steps of 50 N for every 10,000 cycles, the test was carried out until failure was detected or a threshold of 1,500 N was reached; in case of survival up to this step (1,500 N), the step was increased to 100 N for every 10,000 cycles, until failure or test completion at 2,800 N. 32 After finishing each testing step, the specimens were transilluminated to look for potential cracks or fractures. Fatigue failure load (FFL) and number of cycles for failure (CFF) were collected for statistical purposes. Fractography was executed, first using a stereomicroscope to define the representative failure pattern of each group, which was sputter-coated with gold, and later further analyzed in a scanning electron microscopy (VEGA-3G; Tescan, Brno, Czech Republic) at secondary electrons mode, with 20 kV, under 30× and 200× magnification, for crowns and endocrowns, respectively.

Complementary finite element analysis (FEA) was performed to simulate and map stress concentration at the mean FFL observed for each restorative setup during the fatigue test. Three-dimensional models were created, replicating the tested groups while considering the Young's modulus (E) and Poisson's ratios (v) of each material: LD E = 95 GPa, v = 0.25; RC E = 11.61 GPa, v = 0.3; tooth preparation in fiberglass-reinforced epoxy resin E = 18 GPa, v = 0.3; resin cement E = 7.5 GPa, v = 0.3. All solids were assumed to be isotropic with linear behavior, and all contacts were considered perfectly bonded. After meshing the models, the setup was fixed at the same points corresponding to the *in vitro* tests, and the mean FFL values (in Newton) recorded from the *in vitro* tests were applied to each model. The maximum principal stresses were measured (in MPa), and the regions of concentration were mapped in a two-dimensional illustration.

After assuring parametric and homoscedastic distribution, the two-way analysis of variance (ANOVA) and Tukey's post hoc tests were employed, using Statistix 10 (Analytical Software, Tallahassee, United States), with *α* = 0.05, for marginal gap, internal gap, and interfacial volume outcomes. Fatigue data (FFL and CFF) was submitted to survival analysis by means of Kaplan–Meier with log-rank (Mantel–Cox) tests, at the IBM SPSS Software v.21 (IBM, New York, United States), with *α* = 0.05. FEA data was descriptively analyzed.

## Results

Considering the marginal gap, two-way ANOVA indicates that the factor “design” was not statistically significant, meanwhile the factor “material” and the associated factors “design*material” were ( Table 2 ). It can be noted that the lower gap was obtained with the RC crown, while the larger gap was verified in the LD crown. Endocrowns showed intermediate gap values, with resin-based restorations presenting lower gaps than lithium disilicate ones ( Table 3 ).

**Table 2:** Two-way ANOVA tables for marginal gap, internal gaps (cervical, axio-occlusal, occlusal regions), interfacial volume, and fatigue data

**Marginal gap**						**Cervical gap**					
**Source**	**DF**	**SS**	**MS**	***F***	***p*** **-Value**	**Source**	**DF**	**SS**	**MS**	***F***	***p*** **-Value**
**Design**	1	2595	2595	1.05	0.31	Design	1	144075	144075	77.02	0.00
**Material**	1	184946	184946	74.86	0.00	Material	1	1888	188	1.01	0.32
Design* material	1	55513	55513	22.47	0.00	Design*material	1	1172	1172	0.63	0.43
**Error**	476	1175925	2470			Error	476	890369	1871		
**Total**	479	1418978				Total	479	1037504			
**Axio-occlusal gap**						**Occlusal gap**					
**Source**	**DF**	**SS**	**MS**	***F***	***p*** **-Value**	**Source**	**DF**	**SS**	**MS**	***F***	***p*** **-Value**
**Design**	1	165680	165680	43.56	0.00	Design	1	166690	166690	15.81	0.00
**Material**	1	7362	7362	1.94	0.16	Material	1	59630	59630	5.65	0.02
Design* material	1	34301	34301	9.02	0.00	Design*material	1	129875	129875	12.31	0.00
**Error**	716	2723079	3803			Error	236	10546	10546		
**Total**	719					Total	239				
**Interfacial volume**						**FFL/CFF**					
**Source**	**DF**	**SS**	**MS**	***F***	***p*** **-Value**	**Source**	**DF**	**SS**	**MS**	***F***	***p*** **-Value**
**Design**	1	0.23	0.23	0.009	0.93	Design	1	68062.50	68062.50	1.22	0.28
**Material**	1	59.48	59.48	2.316	0.14	Material	1	19113062.50	19113062.50	343.65	0.00
Design* material	1	10.48	10.48	0.408	0.53	Design*material	1	14062.50	14062.50	0.25	0.62
**Error**	36	924.44	25.68			Error	36	2002250	55618.06		
**Total**	40	19488.57				Total	40	1171142500			

Abbreviations: ANOVA, analysis of variance; CFF, cycles for failure; DF, Degrees of Freedom; FFL, fatigue failure load; MS, Mean Square; SS, Sum of Squares.

**Table 3:** Mean, standard deviation (SD), and 95% confidence interval (95 CI) of marginal gap, cervical-axial angle, axio-occlusal angle, and occlusal/pulpal space adaptation values (in µm) and of the volume of the adhesive interface (in mm ^3^ )

Groups	Marginal gap		Cervical-axial angle		Axio-occlusal angle		Occlusal/Pulpal space a		Interfacial volume	
	Mean (SD)	95% CI	Mean (SD)	95% CI	Mean (SD)	95% CI	Mean (SD)	95% CI	Mean (SD)	95% CI
**LD-C**	113.6 (78.8) ^A^	99.3–127.9	136.5 (43.7) ^B^	128.5–144.4	155.7 (51.6) ^B^	146.3–165.0	185.0 (76.2) ^B^	165.1–204.9	22.1 (5.4) ^A^	18.2–26.0
**RC-C**	52.7 (21.2) ^D^	48.8–56.5	136.7 (33.7) ^B^	130.6–142.9	177.0 (51.6) ^A^	167.8–186.3	262.3 (76.2) ^A^	242.9–281.7	20.7 (2.5) ^A^	18.9–22.5
**LD-E**	95.6 (40.5) ^B^	88.2–102.9	167.7 (42.4) ^A^	160.0–175.4	138.0 (77.2) ^BC^	128.1–147.8	283.9 (174.3) ^A^	238.5–329.3	23.3 (7.5) ^A^	17.9–28.7
**RC-E**	78.4 (39.3) ^C^	71.3–85.5	175.0 (51.7) ^A^	165.5–184.3	130.1 (53.5) ^C^	123.3–136.9	268.7 (33.9) ^A^	259.8–277.6	19.8 (10.4) ^A^	17.5–22.1

Abbreviations: ANOVA, analysis of variance; C, crown; E, endocrown; LD, lithium disilicate; RC, resin composite.

Note: Distinct uppercase letters in each column indicate statistical differences according to two-way ANOVA test with Tukey's post hoc ( *α* = 0.05).

a Occlusal space was considered at crowns, and compared with the pulpal space on endocrowns, which are the interfacial surfaces parallel to the occlusal surface.

With regards to internal gap outcomes, it was seen that the factor “design” was statistically significant for all regions (cervical gap, axio-occlusal gap, and occlusal gap), the factor “material” was statistically significant only for occlusal gap, and the associated factors “design*material” were statistically significant for axio-occlusal gap and occlusal gap ( Table 2 ). Another important aspect that should be noted is that although a space of 120 µm was standardized during restoration planning, all internal regions exceeded this threshold. At the cervical-axial angle, the lowest gap was seen at crowns, regardless of material; even though no difference was found between LD and RC for both crowns and endocrowns. At the axio-occlusal angle, LD crowns presented a lower gap than RC, but for endocrowns, there was no difference between LD and RC. When comparing occlusal/pulpal space, LD crowns showed the lowest values, and RC-C, LD-E, and RC-E were statistically similar ( Table 3 ). For interfacial volume, there was no statistical influence for any of the factors, or when they were considered in association ( Tables 2 and 3 ).

**Fig. 2:**
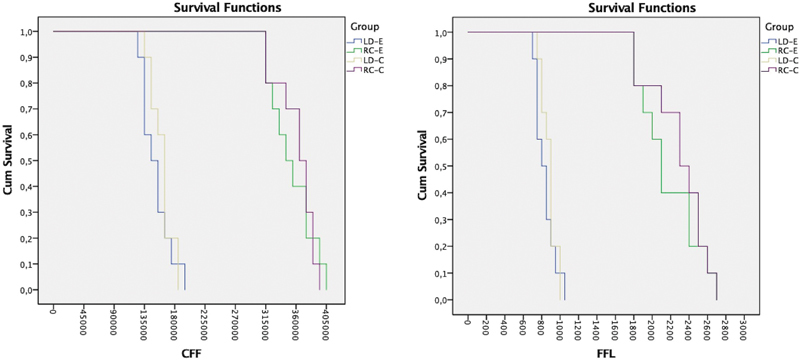
Survival plots of the different tested conditions considering fatigue failure load (FFL – in Newton) and number of cycles for failure (CFF). It is noticeable that lithium disilicate restorations presented a higher survival rate compared to resin composite, regardless of the restorative design.

For fatigue outcomes (FFL and CFF), only the factor “material” showed statistical influence whereas RC restorations were superior to LD ones, independently of the restoration design ( Tables 2 and 4 ). It can be noted that when using LD restorations (both crowns and endocrowns) there is some risk of failure when the applied load surpasses 700 N or 125,000 cycles ( Table 5 and Fig. 2 ). Besides, LD restorations presented a 100% failure rate when they reached loads of 1,000 N or 185,000 cycles. RC restorations required at least 1,800 N or 315,000 cycles to start to show any failure risk (20%), requiring loads above 2,000 N for at least 335,000 cycles to present a higher than 50% risk of failure ( Fig. 2 ).

**Table 4:** Mean, standard deviation (SD), and 95% confidence interval (95 CI) of fatigue failure load (FFL, in Newton), and number of cycles for failure (CFF)

Groups	FFL a		CFF a		Stress calculated through FEA at the mean FFL
	Mean (SD)	95% CI	Mean (SD)	95% CI	Maximum principal stress (MPa)
**LD-C**	888 (82) ^B^	825–951	162,000 (16,414) ^B^	150,160–175,395	215.4
**RC-C**	2255 (296) ^A^	2,027–2,483	360,000 (29,627) ^A^	337,781–383,329	547.0
**LD-E**	850 (103) ^B^	770–929	155,000 (20,615) ^B^	139,153–170,846	59.8
**RC-E**	2133 (308) ^A^	1,896–2,370	349,000 (30,867) ^A^	325,717–373,171	61.7

Abbreviations: ANOVA, analysis of variance; C, crown; E, endocrown; FEA, finite element analysis; LD, lithium disilicate; RC, resin composite.

Note: The maximum principal stress (in MPa) calculated through finite element analysis (FEA) at the mean FFL is presented.

a Distinct uppercase letters in each column indicate statistical differences according to the Kaplan–Meier log-rank (Mantel–Cox) test ( *α* = 0.05).

**Table 5:** Survival rates, that is, specimens' probability to exceed the respective fatigue failure load (FFL, in Newton), and number of cycles for failure (CFF), with their respective standard error measurements

Groups	FFL (N)/CFF																				
	100/5,000	…	700/125,000	750/135,000	800/145,000	850/155,000	900/165,000	950/175,000	1,000/185,000	1,050/195,000	…	1,800/315,000	1,900/325,000	2,000/335,000	2,100/345,000	2,200/355,000	2,300/365,000	2,400/375,000	2,500/385,000	2,600/395,000	2,700/405,000
LD-C	1	…	1	0.9 (0.1)	0.7 (0.2)	0.6 (0.2)	0.2 (0.1)	…	0.0	–	–	–	–	–	–	–	–	–	–	–	–
RC-C	1	…	1	1	1	1	1	1	1	1	…	0.8 (0.1)	…	…	0.7 (0.2)	…	0.5 (0.2)	0.4 (0.2)	0.2 (0.1)	0.1 (0.1)	0.0
LD-E	1	…	0.9 (0.1)	0.6 (0.2)	0.5 (0.2)	0.3 (0.2)	0.2 (0.1)	0.1 (0.1)	…	0.0	–	–	–	–	–	–	–	–	–	–	–
RC-E	1	…	1	1	1	1	1	1	1	1	…	0.8 (0.1)	0.7 (0.2)	0.6 (0.2)	0.4 (0.2)	…	…	0.2 (0.1)	…	0.1 (0.1)	0.0

Abbreviations: C, crown; E, endocrown; LD, lithium disilicate; RC, resin composite.

Note: The sign “-” indicates absence of specimen tested on the respective step. The sign “…” indicates absence of failure on the respective step.

**Fig. 3:**
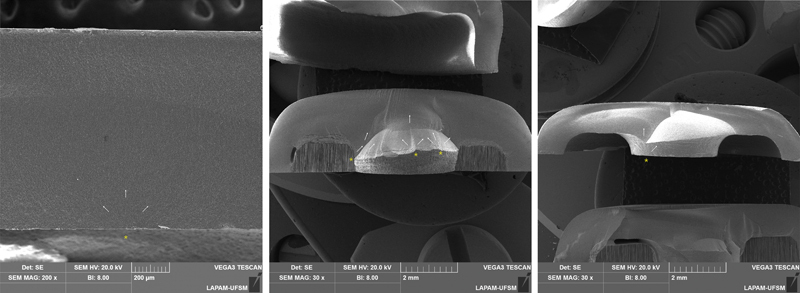
Representative fractographic analysis of the restorations, according to their design and respective materials. Lithium disilicate (LD-C and LD-E) and resin composite (RC-C, and RC-E). The yellow asterisk indicates the failure origin in the crowns' bonding surface, and at the endocrowns pulpal angle, while white arrows indicate the direction of crack propagation.

With regards to the pattern of failure, crowns, regardless of material (LD or RC), fractured from surface defects located at the restoration/cement intaglio surface, which cracks then propagated onto the top, occlusal, opposite surface ( Fig. 3 ). As for endocrowns, the crack initiated at surface defects located at the restoration/cement intaglio surface juxtaposed to the pulpal axial angle, at the entrance of the pulpal chamber where the restoration prolonged itself. Such cracks then propagated toward the occlusal surface.

**Fig. 4:**
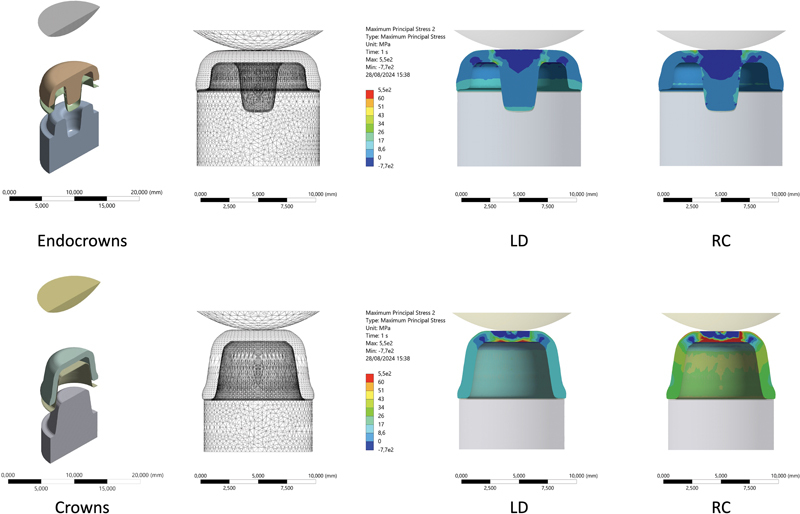
Representative images of the finite element analysis (FEA) simulating stress concentration at the mean fatigue failure load observed for each restorative setup during the fatigue test. The first image on the left shows an exploded view of each restorative setup, including tooth preparation, the cement layer, restoration, and load applicator. The second image from the left displays the mesh for each restorative design. The two images on the right present the FEA results (stress distribution) for the two restoration designs (endocrowns at the top and crowns at the bottom) and the two restorative materials (lithium disilicate – LD, on the left; and resin composite – RC, on the right).

FEA data ( Fig. 4 and Table 4 ) showed that the amount of stress concentration required to unleash the restoration failure varies between restorative materials and designs, whereas crowns required more stress concentration to unleash their failure than endocrowns. Besides that, RC crowns required also more stress concentration to unleash their failure than LD ones. The regions where stress concentrated also differed among restorative designs. In endocrowns, the maximum stress was observed at the pulpal angle between the occlusal surface of the tooth preparation and the entrance to the pulpal chamber. For crowns, the maximum stress was seen on the occlusal surface, a few millimeters lateral to the center of the crown. The material factor did not influence this aspect.

## Discussion

The present findings revealed that the design factor significantly influenced all internal gap regions (cervical, axio-occlusal, and occlusal gaps). The material factor impacted marginal gap and fatigue outcomes (FFL and CFF), while the interaction between design and material factors influenced marginal gaps and some internal gap regions (axio-occlusal and occlusal gaps). Consequently, the study's null hypotheses 1, 2, and 4—asserting no influence of design and material on these outcomes—were rejected. Only null hypothesis 3, suggesting that fatigue behavior is not affected by restoration design, was accepted.

It is established that various factors during restoration manufacturing can affect its fit to the tooth preparation. 16 17 33 34 35 36 37 Distortions can occur during tooth impression; however, literature shows that intraoral scanners have advanced to a level where they match or surpass traditional impression techniques. 38 39 40 In this study, the CEREC Primescan (Dentsply Sirona) was used for direct digitalization of the preparations. This scanner employs both active triangulation and confocal microscopy, aligning with existing literature that suggests optimal accuracy is achieved when multiple imaging principles are used. Active triangulation estimates object position based on the known positions and angles of two other points, while confocal microscopy correlates object position and distance with the focal length of the lens. 35 Therefore, potential distortions related to the tooth impression and digitalization process were likely minimal, representing the best possible current performance for such procedures.

Despite this, it is important to note that the planned cement space during restoration was set at 120 µm, a threshold traditionally accepted as adequate for maintaining clinically acceptable biological responses in surrounding tissues. 18 May et al 21 further demonstrated that the benefits of an adhesive cementation strategy, which enhances the reinforcement of ceramic restorations, are only realized when the internal gap remains below 300 μm, with thinner cement intaglio surfaces optimizing performance. Studies directly correlating the planned space, and the actual gap post-CAD-CAM milling are scarce, indicating a need for further exploration. 33 41 42 In this study, RC restorations exhibited smaller marginal gaps compared to LD restorations; however, no clear trend was observed when comparing the internal gaps between the two materials, suggesting that there is the influence of other variables evolved. Nonetheless, it is noteworthy that in this study, internal gaps, although larger than planned ( Table 3 ), remained within the 300-μm threshold, 21 and marginal gaps were consistently below the 120-μm threshold. 18 These findings suggest that the variations observed in marginal and internal gaps are likely clinically insignificant ( Table 3 ) and clinical decisions regarding material and design may not be primarily influenced by these outcomes, though this conclusion should be cautiously considered given the limitations of this *in vitro* study.

Another factor potentially influencing marginal and internal gaps is distortion during the CAD-CAM milling process. 33 43 Milling accuracy is affected by the characteristics of the burs used, the milling modes (e.g., slow or fast), the number of burs employed, the number of axes in the milling system, and the material being milled. 33 43 44 45 46 A recent scoping review indicated that accuracy and precision between planned and final restoration dimensions are optimized when finer burs, longer milling times, and machines with more axes are used. 33 In this study, we utilized the CEREC MC XL (Dentsply Sirona), a 4-axis machine operating at 42,000 rpm with two burs (bur 12S and Cylinder pointed bur 12S, that reaches 1.00 mm, with 65 μm grit size). Despite being a 4-axis system, CEREC MC XL is one of the most used, accurate, and precise systems observed in scientific literature. 33 45 Thus, we believe that any potential distortions related to the milling process were minimal and consistently distributed across the groups, with variations attributed solely to the study design and the factors considered within it.

With regard to the different characteristics within the existent material options for CAD-CAM processing, the LD material is composed of lithium disilicate crystals, larger than 1 µm and needle-like in shape, randomly distributed within a vitreous matrix at a 70% volume ratio. In contrast, RC consists of a cross-linked dimethacrylate matrix filled with barium aluminum silicate glass particles (< 1 µm in size) and silicon dioxide fillers (< 20 nm in size), also with a filler content of approximately 70% by volume. Despite the similarity in filler volume ratios, the microstructural differences between these materials lead to distinct mechanical behaviors under load. RC exhibits a lower elastic modulus and greater resilience, whereas LD, as a ceramic material, is more brittle and incapable of sustaining plastic deformation. 47 48 49 50 This characteristic leads to RC being more easily milled than LD, which cannot be directly understood of something beneficial. Furthermore, it is true that LD milling has being classified as hard milling, which is known to induce a cascade of events on the ceramic surface and subsurface resulting in radial and lateral cracks, chipping, damage, and residual stress introduction, 25 28 51 52 and that all of these factors constitute potential sites for fracture initiation and consequent failure of the respective restoration in a clinical environment. 26 27 28 51 53 Despite that, RC are so less resistant than LD that the system can, in some regions, generate CAD-CAM overmilling, thus removing unplanned material regions and causing distortions from what was initially planned. 33 41 Hence, we believe that the reasons for the greater performance of RC over LD is basically microstructural differences, higher resilience of RC, lesser brittleness, and enhanced compatibility between restorative material and substrate, which present more similar elastic modulus, 47 48 49 50 54 summed to lesser damage during milling, with lesser residual stresses being incorporated into the material structure. 15 33 Moreover, RC has an enhanced bonding performance to the resin cement, inducing enhanced stress distribution through the restorative set. 55

Our findings indicate that RC restorations exhibited lower marginal gap, although thicker gaps were observed in the occlusal/pulpal space, likely due to overmilling. Similar overmilling/distortions were observed in endocrowns within the pulpal chamber, where scanner accuracy decreases and milling becomes more challenging, fact that results in larger cement intaglio thicknesses. 15 30 33 56 Besides misfit, the potential of milling in introducing surface defects and residual stresses should also be considered, which could facilitate crack propagation when the restoration is submitted to the oral functional stimuli afterwards. 25 28 51 52 Even so, adhesive cementation may heal surface defects induced during milling and optimize mechanical performance of such restoration. 55

The condition of tooth structure is a major point to consider and to give this substrate an adequate form is a critical step. Errors in tooth preparation, or insertion axis during placement, 29 37 or neglecting fundamental principles of tooth preparation (e.g., appropriate wall convergence and/or parallelism, preparation height, rounded angles, and adequate finish line), 57 can increase cement thickness, leading to potential failures. Larger cement surfaces increase the likelihood of critical defects, stress concentration, and premature failure (Griffith law). 58 Studies have shown that increased cement thickness also increases air bubbles, reduces adhesion, increases cement solubility, and compromises long-term performance. 59 60

Our findings revealed that fatigue behavior was influenced solely by the restorative material, with RC outperforming LD ( Tables 2 , 4 , and 5 ). RC's lower brittleness allows it to endure higher loads and better distribute stresses onto the remaining tooth structure. 47 48 49 50 According to the manufacturer and corroborated by the literature, 61 the flexural strength of the RC used in this study (Tetric CAD) is approximately 272 MPa, while lithium disilicate (IPS e.max CAD) is around 530 MPa. 50 The reduction in strength observed between these literature values and the FEA results supports the slow crack growth mechanisms triggered by fatigue testing, which induce cumulative damage. 48 62 This mechanism is logical and directly related to the brittleness of ceramic materials. 47 49 It is further supported by fractography analysis, which shows failures initiating at the cement intaglio surface ( Fig. 3 ), where the FEA indicates maximum stress concentration.

The performance of RC in endocrowns also aligns with this fatigue mechanism, and FEA confirms the regions where fractures begin. Furthermore, RC crowns demonstrated optimal performance, likely due to the resin composite's elastic modulus, which is more compatible with the tooth structure, allowing greater deformation without causing fractures and improving stress distribution throughout the restorative assembly. 48 62 Additionally, the enhanced support provided by the tooth preparation for crowns likely contributes to their superior performance compared to endocrowns in terms of requiring more stress concentration to unleash fracture as shown by the FEA data. 63 64 An aspect that could have influenced such behavior is the fact that the area of contact between the crown and the support tooth is external, meanwhile endocrowns has its prolongation into the pulpal chamber, thus distributing the load that is applied onto it at the long axis of the whole teeth. Such biomechanics could be responsible for the differences of stress distribution considering the restorative design factor, showing optimized performance of endocrowns in distributing the stress than crowns, which denoted more stress concentration.

With regard to the FFLs endured by the restorative setup and clinical function requirements, it is important to note that the observed failure loads far exceeded the normal functional loads typically seen in clinical settings, where the mean chewing load values range between 285 and 462 N for men and between 254 and 446 N for women. 65 66 They were also higher than parafunctional loads seen in bruxist patients, which can reach 627 N. 67 Following this point of view, it seems a good option for clinicians to opt for RC restorations, over LD, when rehabilitating bruxist patients.

A previous report assessed stress distribution in full crowns and endocrowns, finding that endocrowns reduced tensile stress under axial loads, while full crowns performed better under oblique loads. 68 Similarly, the present study applied axial loads, however, the design was not as significant as the restorative material, probably due to the specimen's shape, which allowed a less aggressive contact area than when using anatomical crowns as the previous study. Despite that, both studies corroborate to show less stress concentration in the restoration when a flexible restorative material is used instead of LD.

Finally, this *in vitro* study has inherent limitations, such as using only one scanner and a CAD-CAM system, both of which can impact the final restoration dimensions. Additionally, the anatomy of the occlusal surface was simplified to induce more stability during the fatigue testing; on the other hand, a simplified anatomy also induces enhanced support for the restorations. Thus, the multidirectional incidence of loads resulting from complex occlusal anatomy may influence the results seen herein. Lastly, the resin cement used had a similar composition to one of the restorative materials (resin composite), potentially affecting the accuracy of computed microtomography analysis. Despite these limitations, this study successfully compared and characterized the performance of different restoration designs and materials in terms of marginal gaps, internal gaps, interfacial volume, and their correlation with mechanical fatigue performance, supporting the adequacy and similar performance of different restorative designs.

## Conclusion

Resin composite, crowns, and endocrowns, present smaller gaps at the restoration margins; differences in other internal gap outcomes exists, but within a potential nonrelevant threshold.

The fatigue performance was not influenced by the restorative design; namely, crowns and endocrowns showed a similar FFL, CFF, survival probabilities, and pattern of failure (fractographical features). Despite that, crowns required more stress concentration to unleash their failure than endocrowns.
